# Synergistic Antibacterial and Anti-Inflammatory Activity of Temporin A and Modified Temporin B In Vivo

**DOI:** 10.1371/journal.pone.0007191

**Published:** 2009-09-28

**Authors:** Rosanna Capparelli, Alessandra Romanelli, Marco Iannaccone, Nunzia Nocerino, Raffaella Ripa, Soccorsa Pensato, Carlo Pedone, Domenico Iannelli

**Affiliations:** 1 Department of Soil, Plant, Environment and Animal Production Sciences, University of Naples “Federico II”- School of Biotechnological Sciences, Napoli, Italy; 2 Department of Biological Sciences, University of Naples “Federico II”- School of Biotechnological Sciences, Napoli, Italy; Massachusetts General Hospital and Harvard Medical School, United States of America

## Abstract

Temporins are antimicrobial peptides secreted by the granular glands of the European red frog (*Rana temporaria*). They are 10–14 amino acid long polypeptides active prevalently against gram positive bacteria. This study shows that a synthetic temporin B analogue (TB-YK), acquires the capacity to act in synergism with temporin A and to exert antimicrobial and anti-inflammatory activity in vivo against gram positive and gram negative bacteria. Administration of 3.4 mg/Kg of temporin A (TA)+1.6 mg/Kg TB-YK, given to individual mice concurrently with a lethal dose of bacteria (gram positive or negative), rescued 100% of the animals. More importantly, the same doses of temporins, administered one week after experimental infection with a sub lethal dose of bacteria, sterilized 100% of the animals within 3–6 days. Also, it is described an animal model based on the use of sub lethal doses of bacteria, which closely mimics bacterial infection in humans. The model offers the possibility to test in a preclinical setting the true potential of TA and TB-YK in combination as antimicrobial and anti-inflammatory agents.

## Introduction

Antimicrobial peptides (AMP) are essential components of the innate immune system of plants and animals, including humans [1]. AMPs, though highly different in size and secondary structure, display important common characteristics, such as a net positive charge at neutral pH, the ability to interact with the bacterial membrane and kill a large spectrum of bacteria [2]. The worldwide phenomenon of bacterial resistance to conventional antibiotics has made urgent the search for antibacterial drugs active against antibiotic-resistant bacteria [3]. AMPs are among the most promising candidates, in view of their small size (which simplifies their chemical synthesis), a large spectrum of activity against gram positive and gram negative bacteria, and limited toxicity towards eukaryotic cells [4], [5]. In particular, temporins are AMPs produced and secreted by the granular glands of the European red frog (*Rana temporaria*) [6], [7]. They are amphipathic α-helical polypeptides, 10–14 amino acid long, containing only 1 or 2 positively charged amino acids (R or K). These peptides are amidated at the C-terminus as a result of an enzymatic post-translational reaction [8]. Temporins are active prevalently against gram positive bacteria, including methicilline and vancomycine-resistant staphilococci and enterococchi, and are non toxic on mammalian cells. Temporin L, which contains an arginine and a lysine residue in the sequence, is the only member of the family which exhibits antibacterial activity against both gram positive and negative bacteria [9]. Interestingly temporins A and B, in combination with Temporin L, show anti-bacterial activity against Gram negative bacteria [10]. The mechanism of action of temporins has not entirely been clarified; it has been shown that the sequence, the charge distribution, the secondary structure all influence the ability of the peptides to interact with bacterial membranes; cationic AMPs preferentially interact with the negatively charged bacterial membranes by altering the membrane permeability [11], [12]. In Gram negative bacteria the microbial wall is surrounded by the lipopolysaccharide layer (LPS), which represents a further hurdle for the peptides before the cytoplasmic membrane. It has been demonstrated that Temporins A and B oligomerize when they reach the LPS, suggesting that the inactivity of these peptides towards Gram negative bacteria may be due to the oligomerization process which in turn hampers the peptide uptake [10]. In contrast, Temporin L disaggregates when in contact with the LPS; furthermore it is also able to inhibit oligomerization of temporin A and B, allowing for the translocation of these peptides in the Gram negative inner membrane. Data recently reported on AMPs derived from a combinatorial library suggest that the antimicrobial activity of AMP is related to the ability of the peptide to destabilize membranes by partitioning into membrane interfaces and disturbing the organization of the lipids [13].

Few analogues of temporins have been investigated so far [14]–[17]. Dimeric Temporin A was shown to have antimicrobial activity also towards both the Gram negative bacteria *E.coli*, unlike the wt Temporin A [16]. The temporin A P3N analogue exhibits improved ability to lyse human red blood cells as compared to wt Temporin A and an improved antimicrobial activity especially against Gram-positive bacterial strains and Candida [15]. In vitro experiments showed that the D isomer of Temporin B is active against several anaeorobic bacteria and is also stable in the fecal milieu, suggesting it can be employed for the treatment of the Clostridium difficile. [17] The effect of Temporin A was also studied *in vivo* in a mouse model of Staphylococcus sepsis; it was found that the peptide reduces the lethality rates and bacterial growth, the antibacterial activity is enhanced when Temporin A was used in combination a with the antibiotic Imipenem [18].

The present article demonstrated by in vitro and in vivo experiments that synthetic temporin A (in its native form) and a temporin B analogue act synergistically against the majority of gram positive and gram negative bacteria tested. Notably, the combination of the two temporins is active against methicillin-resistant strains of *S. aureus*.

## Materials and Methods

### Synthesis of antimicrobial peptides

The amino acids used for the peptide synthesis, Fmoc-β-Ala-OH, Fmoc-Arg(Pbf)-OH, Fmoc-Asn(Trt)-OH, Fmoc-Gly-OH, Fmoc-Ile-OH, Fmoc-Leu-OH, Fmoc-Ser(OtBu)-OH, Fmoc-Phe-OH, Fmoc-Pro-OH, Fmoc-Val-OH, Fmoc-Tyr(OtBu)-OH -, the Rink amide MBHA and the activators N-Hydroxybenzotriazole (HOBT) and O-Benzotriazole-N,N,N',N'-tetramethyl-uronium-hexafluoro-phosphate (HBTU) were from Novabiochem (Gibbstown, NJ, USA). Acetonitrile (ACN) was from Reidel-deHaën (Seelze, Germany) and dry N,N-dimethylformamide (DMF) from LabScan (Dublin, Ireland). All other reagents were from Fluka (Milan, Italy). LC-MS analyses were performed on a LC-MS Thermo Finnigan with an electrospray source (MSQ) on a Phenomenex Jupiter 5 µ C18 300 Å, (150×4.6 mm) column. Purification was carried out on a Phenomenex Jupiter 10 µ Proteo 90 Å (250×10 mm) column. Peptides were synthesized on a Rink-amide 4-methyl benzhydrylamine (MBHA) resin (0.63 mmol/g) using repetitive cycles of coupling, capping and deprotection. Amino acids (10 eq.) were preactivated for 5 min in the presence of a 0.5 M hydroxybenzotriazole (HOBT) and O-benzotriazole-N,N,N,N,-tetramethyl-uronium-hexafluoro-phosphate (HBTU) (9.8 eq) in dimethylformamide (DMF) solution and N,N-diisopropylethylamine (DIPEA) (30 eq) and coupled for 60 minutes. The resin was washed with DMF (two flow washes, 25 s each). Capping was performed with a solution of acetic anhydride/DIPEA/DMF (15/15/70 v/v/v) for 5 min, followed by two flow washes (25 s each) with DMF. Deprotection was performed with a solution of piperidine in DMF (20/80; v/v) for seven minutes. The resin was washed with DMF (two flow washes of 25 s each). At the end of the synthesis, the resin was washed with dichloromethane and dried in vacuum. The peptides were cleaved off the resin and deprotected by a treatment with a solution of trifluoroacetic acid (TFA)/triisopropyl silane (TIS)/H_2_O (90/5/5) (90 minutes at room temperature). Peptides were purified using the following gradient: isochratic 30% water containing 0.1% TFA (5 min), followed by a gradient of 30–80% acetonitrile in water containing 0.1% TFA (30 min). Lyophilized peptides were dissolved in H_2_O/CH_3_COOH (1/1; v/v) and lyophilized again. All peptides were characterized by electrospray mass analysis.

### Peptide labelling

Reactions with fluorophores were carried out on peptides still anchored to the resin. Two β alanines (βA) were added at the N- terminus of TB-YK and TA before labelling. [19] Fluorescein isothiocyanate or rhodamine isothiocyanate (10 eq; Sigma-Aldrich, Milan, Italy) were dissolved in the minimum volume of dry DMF containing DIPEA (30 eq) and incubated for 2 hours at room temperature in the dark with the resin bound peptides. The resin was then washed with DMF, and the peptide cleaved off the resin, deprotected and purified as described above.

### Circular dichroism

Circular dichroism (CD) spectra were recorded on a Jasco J-715 spectropolarimeter, equipped with a Jasco Peltier PTC-423S/15, using a 1 cm quartz cell. Spectra were recorded in 10 mM sodium phosphate buffer pH 7.4 and in 10 mM sodium phosphate, 20 mM sodium dodecyl sulphate (SDS) buffer pH 7.4 at 25°C. Peptides concentration was 5 µM. Spectra were obtained as an average of eight scans, acquired in the range 320–195 nm. All spectra are reported in molar ellipticitiy per residue (mre) vs wavelenght (nm).

### Bacteria

The study included the following species: *Staphylococcus aureus* (isolate A170, A172, 007), *Listeria monocytogenes*, *Salmonella enterica* serovar Paratyphi, *Salmonella enterica* serovar Typhimurium, *Escherichia coli*. Isolates were obtained from patients hospitalized at the Medical School of the University of Naples. Specimen were confirmed by PCR assay of the genes *sea* (*S. aureus*), *MonoA* (*L. monocytogenes*), *abe* (*S. enterica* serovar Paratyphi), *FliC* (*S. enterica* serovar Typhimurium), *sat* (*E. coli*) [20]–[23]. Bacteria were grown at 37°C in TSB medium (*S. aureus* and *L. monocytogenes*) or LB medium (the remaining bacterial species) (Difco, Becton Dickinson, Sparks, MD), harvested while in exponential phase (OD_600_ nm; 0-6-0.8), centrifuged (8×10^3^ g for 10 min), washed with saline (0.15 M NaCl) and resuspended in saline (10^6^–10^7^ CFU/ml).

#### *S. enterica* transformation

The green fluorescent protein (GFP) gene was cloned into the HindIII-PstI sites of the pUC19 plasmid. The plasmid was then introduced into *S. enterica* serovar Paratyphi B (isolate 0404572) by electroporation as described [24]. Briefly, bacteria were grown to mid-log phase in LB broth, washed with 10% glycerol in double distilled H_2_O, suspended at 10^12^ cells/ml and mixed with 1 µg of DNA. An aliquot (40 µl) was pulsed at 1.75 kV/cm, 800 Ω, and 25 µF. Following electroporation, the transformed bacteria were incubated for 30 min at 37°C in 1 ml LB broth supplemented with 0.3 M sucrose. Bacteria were then spread on LB agar plates containing 100 µg/ml ampicillin (Sigma-Aldrich, Milan, Italy).

### Mice

Experiments were carried out on females BALB/c mice (aged 8–10 weeks) at the animal facility of the University of Naples. Untreated mice were infected intravenously with bacteria (*S*. *aureus*: 10^7^ CFU/mouse and *S. enterica* serovar paratyphi: 10^6^ CFU/mouse). Treated mice were infected (as just described) and immediately after were given intravenously the antimicrobial peptide combination (3.4 mg/Kg TA+1.6 mg/Kg TB-YK in saline (0.15 M NaCl). One gram of infected organs - the kidneys in the case of infection with *S aureus*; liver and gastro intestinal (GI) tract in the case of infection with remaining bacteria - was homogenised in 1 ml saline and serially diluted in saline. CFU were evaluated by plating each dilution on Baird Parker agar (to detect the *S aureus*) or XLT-4 agar (to detect the remaining bacteria). Plates were incubated overnight at 37°C.

### Antibacterial activity

Bacteria were grown at 37°C in TSB (*S. aureus* and *L. monocytogenes*) or in LB medium (the remaining bacterial species), harvested while in exponential phase (OD_600_ nm; 0-6-0.8), centrifuged (8×10^3^ g for 10 min), washed with saline, resuspended in Muller Hinton (MH) broth at the concentration of approximately 10^6^ CFU/ml and distributed, in triplicate, into 96 well plates (60 µl/well), mixed with twofold dilutions of the antimicrobial (TA: 5–100 µg, 40 µl/well; TB-YK: 4–100 µg, 40 µl/well; TA 5 µg+TB-YK 8 µg, 40 µl/well and incubated at 37°C for 20 h. The minimal concentration of the antimicrobial at which 100% inhibition of growth was observed (MIC_100_) was determined by measuring the absorbance at 600 nm (Biorad microplate reader model 680, Hercules, CA). The antibacterial activity was measured by spotting an aliquot (10 µl) of each well on TSA or LB agar and counting the CFU. A potential synergism between temporins was evaluated by adding combinations of two temporins in a serial twofold dilutions (TA: 5–100 µg;TB-YK: 4–100 µg; 40 µl/well) to wells containing 10^5^ CFU/well in 60 µl. The presence of synergism was established as described [10].

### Inhibition zone assay

Bacteria were grown as described above, suspended in LB (2×10^5^ CFU/ml), mixed with 3 ml of 0.7% soft agar (Sigma) and poured on Petri dishes containing 10 ml LB agar. Serial dilutions of the antimicrobial (TA and TB-YK) alone or in combination) were deposited into holes (10 µl/well) made into the agarose bed. Plates were then incubated at 30°C for 12 h.

### Electron microscopy

Bacteria (*S. aureus* or *S. enterica* serovar Paratyphi; 10^6^ CFU/60 µl) were mixed on a microscope slide with the antimicrobial combination (*S. aureus*: 5 µg TA+8 µg TB-YK in a total volume of 40 µl; *S. enterica* serovar Paratyphi: 100 µg TA+4 µg TB-YK in a total volume of 40 µl), incubated overnight in 2.5% gluteraldehyde and then treated for 1 h, in succession, with 20%, 40%, 60% ethanol and overnight with 70% ethanol. The slides were finally washed with 90% and 100% ethanol for 1 h, dried at 37°C for 15 min and observed with the SEM Zeiss microscope (Evo 40, Jena, Germany).

### Test of the haemolytic activity of the antimicrobials

The temporins were tested individually for their haemolytic activity using mouse red blood cells. The blood was collected from the tail of the animals and centrifuged (4×10^2^ g for 3 min). The erythrocytes were washed with saline, suspended at 3×10^6^ erythrocytes/ml, mixed with the peptide combination (75 µg TA+35 µg TB-YK in 100 µl saline) and incubated for 1 h at 37°C. The haemolytic activity was measured according to the formula OD peptide - OD _negative control_/OD _positive control_ - OD _negative control_ ×100 where the negative control (0% haemolysis) was represented by erythrocytes suspended in saline and the positive control (100% haemolysis) was represented by the erythrocytes lysed with 1% triton X100 [25].

### Real time PCR of pro-inflammatory cytokines

Two groups of mice (4 mice/group) were infected intravenously with *S. enterica* serovar Paratyphi. One group of mice was used as untreated control, while the second group was injected intravenously with the antimicrobial (3.4 mg/Kg TA+1.6 mg/Kg TB-YK; 75 µg TA+35 µg TB-YK n 100 µL in saline) immediately after infection. Total RNA was isolated from mouse liver at 3, 6, 24 and 48 h after treatment by using Trizol reagent (Invitrogen, Milan, Italy). RNA was suspended in RNase-DNAse free distilled water, assessed for concentration (by measuring the absorbance at 260 nm) and purity (by ascertaining that the A260/A280 ratio was >1.9). RNA (1 µg) was then treated with 1U RNAse-free DNAse (Promega, Madison, WI). DNA contamination of RNA samples was excluded by PCR with primers specific for the *gapdh* gene. Reverse transcription was carried out with ImProm-II reverse transcriptase (Promega, Madison, WI) and oligo(dT)_18_. Real-time PCR was performed on 50 ng cDNA, using 1x master mix SYBR-Green (Applied Biosystem, Milan) in a StepOne Applied Biosystem instrument (Applied Biosystem, Milan). Reactions were performed in 20 µl in triplicate with the following thermal profile: 95°C for 10 min and 45 cycles of 15 s at 95°C and 45 s at 60°C;1 min at 60°C; 15 s at 95°C plus 0.3°C at each step of the melting curve. PCR primers (0.2 µM each) were as follows: GAPDH forward, 5′ TTCACCACCATGGAGAAGGC 3′; GAPDH reverse, 5′ GGCATGGACT GTGGTCATGA 3′; IL-6 forward, 5′ AAAGAGTTGTGCA ATGGCAATT 3′; IL-6 reverse, 5′ CAGTTTGGTAGCATCCATCAT 3′; TNF-α forward, 5′ TCTCAGCCTCTTCTC ATTCCT3′ ; TNF-α reverse, 5′ GTCTGGGCCATAGAACTGATG 3′; IL-4 forward, 5′AATGTACCAGGAG CCATATCCAC 3′; IL-4 reverse, 5′ TCACTCTCTGTGGTGTT CTTCGT 3′; IFÑγ forward, 5′ AGCGGCTGACTGAACTCAGATTGTAG 3′; IFN-γ reverse, 5′ GTCACAG TTTTCAGCTG TATAGGG 3′.

### Other methods

Leukocytes formula and haemachrome of mice were determined with the Am45 instrument (Melet Schloesing, Osny, France). Mice infected with GFP-labelled bacteria were analysed using the Leica macrofluo instrument (Wetzlar, Germany) equipped with the Leica application suite 3.1.0 software. Survival rates of mice were analyzed using Fisher's exact test. Bacterial counts and cytokines levels were analyzed using Student's t test.

## Results

### Synthesis and circular dichroism characterization of the peptides

**Figure 1:**
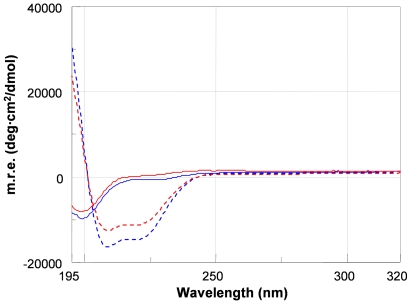
CD spectra of: TB (blue, continuous line), TB-YKβA (red continuous line) in Sodium Phosphate buffer pH 7.4 (-); TB (blue dashed line) and TB-YKβA (red dashed line) in SodiumPhosphate buffer+SDS pH 7.4.

Antimicrobial peptides were synthesised by standard solid phase technique and characterized by LC-MS. Analysis of the temporin B structure suggested some modifications that potentially might have improved its use. First, one extra tyrosine residue was added to the N- terminus of the temporin B. This modification conferred to the peptide a distinctive absorption pattern at 280 nm. The concentration of the peptide could thus be measured precisely. Second, studies of the interaction between the temporin B and biomembrane models have shown that temporin B forms a complex with the acidic phospholipid component of the membrane [26]. The addition of two extra positive charged lysine residues to the N-terminus of the temporin B, to give TB-YK, was therefore thought to facilitate the electrostatic interaction with the negatively charged phospholipids. For the in vivo studies peptides need to be labelled with fluorescent dyes; we obtained temporin A derivatized with rhodamine (TA-Rho) and Temporin B derivatized with a fluoresceine (TB-Fluo). Before labelling, to prevent degradation of the peptide, two β alanines (βA) were added at the N-terminus of temporins [19]. The secondary structure of TB and TB-YKβA analogue was studied by CD in phosphate buffer and phosphate buffer plus SDS. CD spectra of the peptides in sodium phosphate buffer pH 7.4 showed a minimum close to 200 nm, indicative of a random coil conformation. In contrast, in SDS both peptides assumed an α helical conformation, as suggested by the minima around 209 and 222 nm (Figure 1).

### In vitro experiments

**Figure 2:**
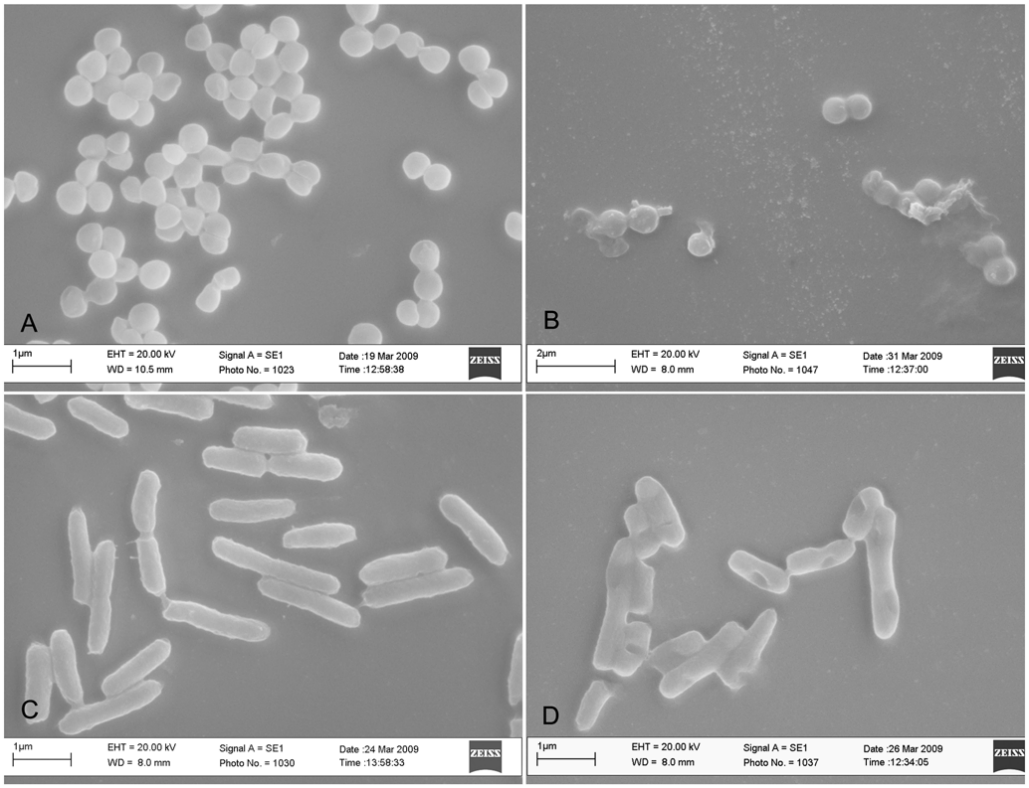
Temporins initiate their antibacterial activity by drillings holes in the bacterial membrane. A: *S.aureus* A170, untreated; B: *S aureus* A 170 treated for 10 min with TA (8 µg/ml) plus TB-YK (5 µg/ml) (B); C: *S.enterica* serovar Paratyphi B untreated; D: *S.enterica* serovar Paratyphi B treated for 10 min with TA (100 µg/ml) plusTB-YK (4 µg/ml).

**Figure 3:**
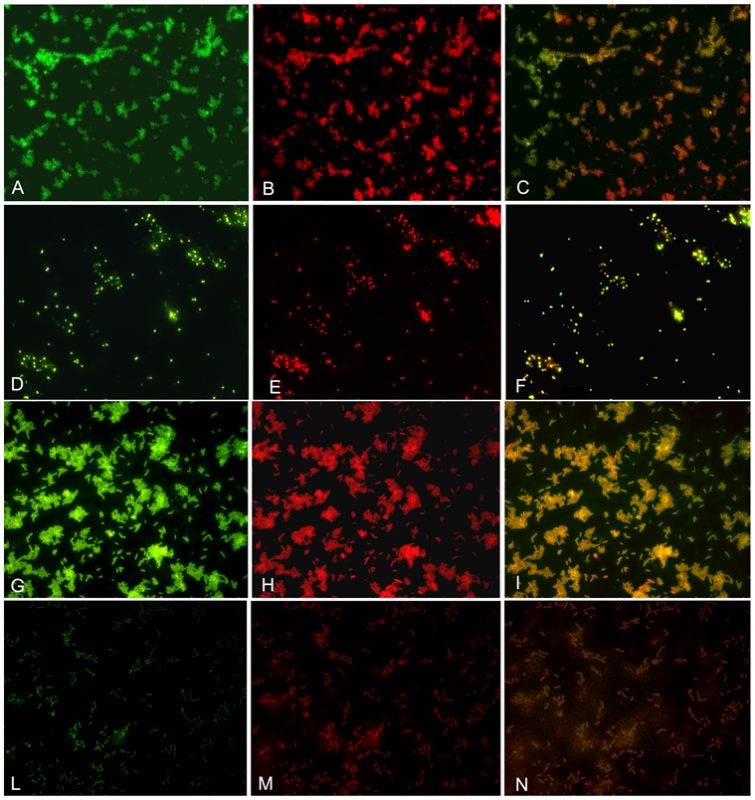
Detection of total, viable and membrane-perturbed bacteria using temporin A or temporin B labeled with rhodamine isothiocyanate or fluorescein isothiocyanate respectively. A- B-C: *S.aureus* A 170 plus TA-Rho 6 µg plus TB-Fluo 9 µg; D-E-F: *S.aureus* A170 plus fluorescein isothiocyanate o rhodamine isothiocyanate (control); G-H-I: *S.enterica* serovar Paratyphi B plus TA-Rho 15 µg plus TB-Fluo 20 µg; L-M-N: *S.enterica* serovar Paratyphi B plus fluorescein isothiocyanate o rhodamine isothiocyanate (control).

**Figure 4:**
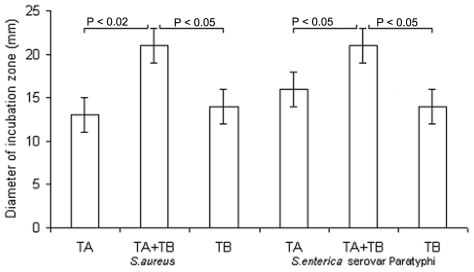
Synergism between TA and TB-YK temporins. The diameter of the inibition was maesured after 24 h incubation.

The study examined the antimicrobial activity of the synthetic temporins A and B in their native forms, and of the temporin B following chemical modifications (Table 1); the latter temporin was studied alone and in combination with native temporin A. The native temporin A was more active against gram positive (MIC: 10–30 µg/ml) than against gram negative (MIC: 125–400 µg/ml) bacteria (Table 2); the native temporin B and TB-Y were active against gram positive bacteria (approximately to the same extent as temporin A), but totally inactive against gram negative bacteria (data not shown). Also, tested in different ratios, the two native temporins did not display synergism. Instead TB-YK exhibited antimicrobial activity either against gram positive and gram negative bacteria (Table 2). Remarkably, when modified TB-YK (at 4–8 µg/ml) was used in combination with the native temporin A (at 5–100 µg/ml), a strong synergism was observed against gram positive as well as against gram positive bacteria (Table 2). Electron microscopy showed the ability of the combination of TA and TB-YK to perforate the bacterial membrane (Figure 2). The labelled peptides, TA-Rho and TB-Fluo, were employed in fluorescence light microscopy experiments. Several combinations of the peptides were tested; the combinations which allowed for a better visualization of the bacteria were 6 µg TA+9 µg TB-YK on *S.aureus 170* and 15 µg TA+20 µg TB-YK on *S.enterica* Paratyphi. The end product of temporins' activity, bacterial fragmentation, is illustrated in Figure 3. An independent experimental approach, the inhibition zone test, confirmed the synergism between the two temporins (Figure 4).

**Table 1:** Amino acid sequence and molecular mass of the temporins used in the study.

Peptide	Amino acid sequence	Molecular	mass
		Calculated	Found
TA	H-FLPLIGRVLSGIL-NH_2_	1396,7	1396,3
TB	H-LLPI VGNLLKSLL- NH_2_	1391.8	1391.2
TB-Y	H-YLLPI VGNLLKSLL- NH_2_	1554.9	1554.7
TB-YK	KKYLLPIVGNLLKSLL- NH_2_	1811,3	1812,8
TB-YKβA	H−βAβA KKYLLPI VGNLLKSLL- NH_2_ (βA = βAla)	1953,4	1953,7
TA Rho	Rho-βAβA KK FLPLIGRVLSGIL-NH_2_ (βA = βAla)	2295,4	2294,3
TB Fluo	Fluo-βAβA KK YLLPI VGNLLKSLL- NH_2_ (βA = βAla)	2342,8	2343,2

**Table 2:** 

Bacterial species/strains	MIC			FIC index^a^
**Gram+**	**TA**	**TB-YK**	**TA+TB-YK**	
*Staphilococcus.aureus* A170	10 µg/ml	20 µg/ml	5 µg/ml+8 µg/ml	0,5
*Staphilococcus .aureus* A172	20 µg/ml	25 µg/ml	8 µg/ml+5 µg/ml	0,5
*Staphilococcus aureus* 007	10 µg/ml	10 µg/ml	8 µg/ml+3 µg/ml	1,1
*Listeria monocytogenes*	30 µg/ml	10 µg/ml	10 µg/ml+3 µg/ml	0,6
**Gram−**				
*Salmonella enterica* serovar Paratyphi	400 µg/ml	20 µg/ml	100 µg/ml+4 µg/ml	0,4
*Escherichian coli*	125 µg/ml	20 µg/ml	50 µg/ml+5 µg/ml	0,6

a The FIC indices were interpreted as follows: ≤0.5, synergy; >0.5, no interaction.

### In vivo experiments

**Figure 5:**
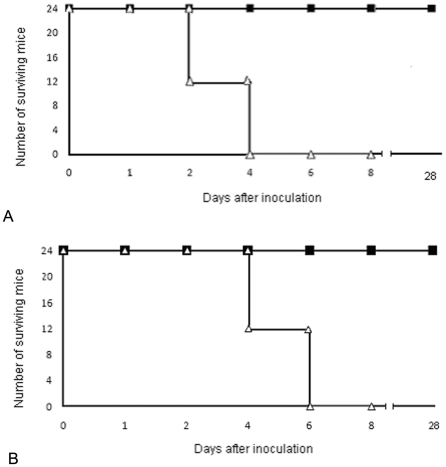
In vivo antibacterial activity of temporins. The 35/75 pool of temporins, given concurrently with bacteria, rescue 100% of mice infected with a lethal dose of *S. aureus* A170 10^7^ CFU/mouse (A: mice infected with *S.aureus* A170 and immediately treated with TA plusTB-YK (closed box) and mice infected with *S.aureus* A170 (open triangle) or a lethal dose of *S. enterica* serovar Paratyphi 10^6^ CFU/mouse (B: Mice infected with *S.enterica* serovar Paratyphi B and immediately treated with TA plus TB-YK (closed box) and mice infected with *S.enterica* serovar Paratyphi B (open triangle)

**Figure 6:**
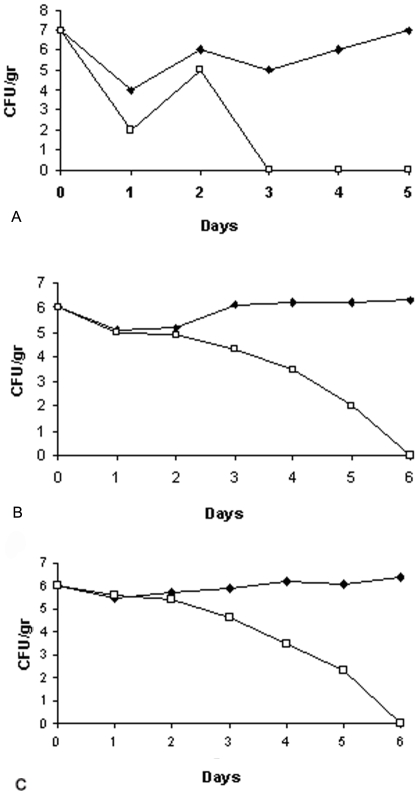
Temporins sterilize mice sperimentally infected with *S aureus* A170 or *S.enterica* serovar Paratyphi B. The 35/75 pool of temporins, given concurrently with bacteria, sterilize mice infected with a lethal dose (10^7^ CFU/mouse) of *S.aureus* A170 (A: kidneys from mice infected with *S. aureus* A170 (closed rhomb) and kidneys from mice infected with *S.aureus* A170 and immediately after treated with TA plus TB-YK (open box), or a lethal dose (10^6^ CFU/mouse) of *S.enterica* serovar Paratyphi B (B: gastro instestinal tract from mice infected with *S.enterica* serovar Paratyphi B (closed rhomb) and gastro intestinal tract from mice infected with *S.enterica* serovar Paratyphi B and immediately treated with TA plus TB-YK (open box); (C: liver from mice infected with *S.enterica* serovar Paratyphi B (closed rhomb) and liver from mice infected with *S.enterica* serovar Paratyphi B and immediately treated with TA plus TB-YK (open box).

On the basis of the results described above, the therapeutic potential of the TA in combination with TB-YK was tested in a mouse model. The test, if successful, offered the possibility to assess the potential clinical use of the two temporins against septic shock caused by gram positive or gram negative bacteria. Several combinations of the two temporins displayed the capacity to protect mice from lethal doses of bacteria. Among these, the combination of 3.4 mg/Kg TA 0.54 mM in saline +1.6 mg/Kg TB-YK 0.19 mM in saline/mouse (75 µg TA+35 µg TB in 100 µl saline) was chosen for further studies because provided the highest surviving rate of mice infected with gram positive and gram negative bacteria and did not display haemolytic activity. This temporin combination, used in all the experiments to be described, will be referred to as the 75/35 pool. Two groups of mice (24 animals/group; untreated groups) were infected intravenously with *S. enterica* serovar Paratyphi (10^6^ CFU/mouse) or *S. aureus* A170 (10^7^ CFU/mouse), respectively. Two more groups of mice (24 animals/group; treated groups) were infected by the same procedure and, immediately after, treated with the 35/75 pool. Untreated mice died all within 4–6 days. The treated mice were all alive 28 days after the temporin treatment. (Figure 5). In an independent experiment, 2 mice infected as described (untreated mice) and 2 mice infected and then treated as above (treated mice) were sacrificed at daily intervals to monitor the bacterial load in the target organs (liver and GI tract of mice infected with *S. enterica* serovar Paratyphi; the kidneys of mice infected with *S. aureus*). Treated mice cleared *S aureus* or *S. enterica* serovar Paratyphi within 3 (Figure 6A) or 6 Figure 6B–C days from temporin treatment, respectively. Untreated mice, instead, remained heavily infected till the time of death (Figure 6A–C). The results demonstrated the efficacy of the 35/75 pool in vivo. Remarkably, the 35/75 pool was active against the methicillin-resistant *S. aureus* A170 strain.

### Efficacy of delayed treatment with the 35/75 pool

**Figure 7:**
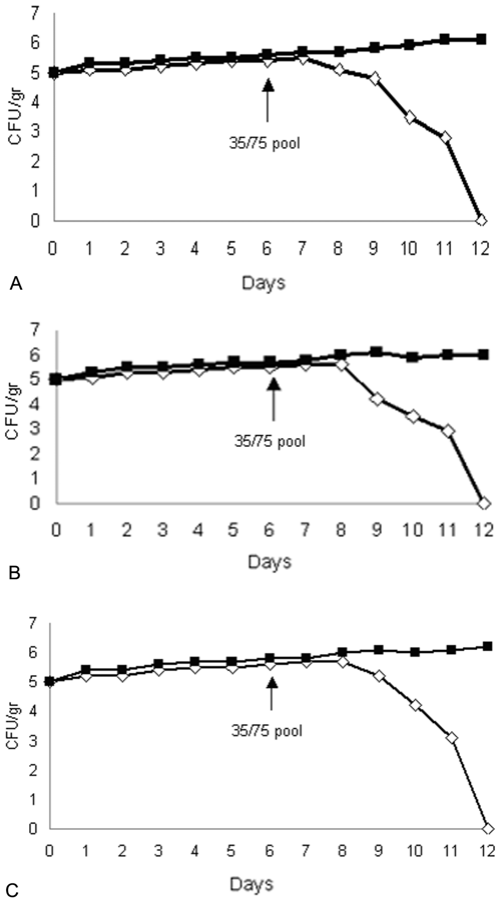
Temporins are effective in vivo when given 7 days after experimental infection. Mice were infected with a sub-lethal dose (10^5^ CFU/mouse) of *S.aureus* A170 (A) or a sub-lethal dose(10^5^ CFU/mouse) of *S.enterica* serovar Paratyphi B (B–C) and 6 days later were treated with the 35/75 pool of temporins. A: kidneys from mice infected with *S.aureus* A170 (closed box) and kidneys from mice infected with *S.aureus* A170 and treated after 6 days with TA plus TB-YK (open rhomb); B: gastro instestinal tract from mice infected with *S.enterica* serovar Paratyphi B (closed box ) and gastro intestinal tract from mice infected with *S.enterica* serovar Paratyphi B and treated after 6 days with TA plus TB-YK (open rhomb); C: liver from mice infected with *S.enterica* serovar Paratyphi B (10^5^ CFU/mouse) (closed box) and liver from mice infected with *S.enterica* serovar Paratyphi B (10^5^ CFU/mouse) and treated after 6 days with the TA plus TB-YK (open rhomb).

Typically, bacterial infections are caused by a small initial inoculum. Therefore, to be clinically useful, temporins must be effective when started several days after infection. To see whether the 35/75 pool displayed this potential, mice were infected with a sub-lethal dose of bacteria (10^5^ CFU/mouse) and one week later treated with the 35/75 pool. The 10^5^ CFU/mouse dose was the inoculum that the innate immune system of the animal could not rapidly control. Again, the pool was effective in sterilizing the animals within 5 days (Figure 7).

### The peptide pool protects from inflammation

Mice infected with *S. enterica* serovar Typhimurium (10^6^ CFU/mouse; untreated mice), 24 hours after infection displayed enlarged spleen, liver, and mesenteric lymph nodes because of inflammation, while mice infected with the same pathogen and immediately after treated with the 35/75 pool (treated mice) displayed only minor morphological changes in the organs. Untreated mice displayed also significant changes of the following parameters, indicative of a systemic immune response to infection: reduced number of red blood cells and leukocytes, reduced haemoglobin concentration and red blood cell volume, (Table 3). Elevated body temperature and elevated levels of circulating lipopolysaccharide (LPS)-binding protein and serum amiloid A (data not shown) characterized the untreated (but not the treated) mice.

**Table 3:** Haematological parameters of mice naive (no infected or treated), infected with *S.enterica* serovar Paratyphi B (10^6^ CFU/mouse), or infected and treated with temporin A(75 µg/mouse) plus temporin B (35 µg/mouse).

	Naive	Untreated	Treated
Leukocytes	8,29±0,56 m/mm^3^	1,23±3,5 m/mm^3^	3,43±0,56 m/mm^3^
Lymphocytes	26,6±2,4%	38,1±1,1%	51,9±1,8%
Monocytes	13,5±0,5%	12,7±0,4%	20,2±1,5%
Neutrophils	59,9±0,9%	40,8±0,6%	26,5±1,26%
Eosinophils	0%	8,1±0,5%	0,8±0,1%
Basophils	0%	0,3±0,6%	0,6±0,3%
Erytrocytes	11,57±0,7 m/mm^3^	10,93±1,7 m/mm^3^	7,63±2,2 m/mm^3^
MCV(mean corpuscolar volume)	43,8±2,7 fl	46,8±2,2 fl	48,3±4,4 fl
HCT (hematocrit)	50,6±4,3%	51,1±4,2%	36,8±3,1%
MCH (mean concentration haemoglobin)	12,7±1,9 pg	12,6±2,5 pg	11,9±1,2 pg
MCHC(mean corpuscolar haemoglobin concentration	29±2,1 g/dl	27±3,1 g/dl	24,7±2,8 g/dl
RDW(widht distribution red cells)	10,4±2,4%	10,9±0,5%	19,7±2,2%
Hb(hemoglobin)	14,7±1,9 g/dl	13,8±1,8 g/dl	9,1±0,9 g/dl
Platelets	482±15,2 m/mm^3^	611±13,7 m/mm^3^	335±19,7 m/mm^3^
MPV(mean volum platelets)	6,1±0,3 fl	5,8±0,9 fl	6,8±1,1 fl
PDW(widht distribution platelets)	7,2±0,6%	7,2±0,7%	7,2±1,2%

P<0,05.

**Figure 8:**
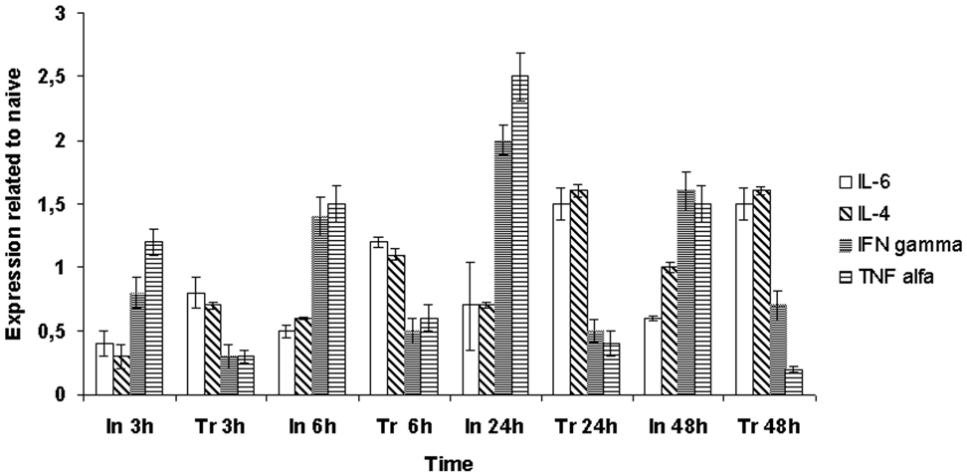
Expression levels of cytokine genes in mice infected with *S.enterica* serovar Paratyphi B (10^6^ CFU/mouse) (In) or infected with *S.enterica* serovar Paratyphi B (10^6^ CFU/mouse) and immediately after treated with the 35/75 pool of temporins (Tr).

**Figure 9:**
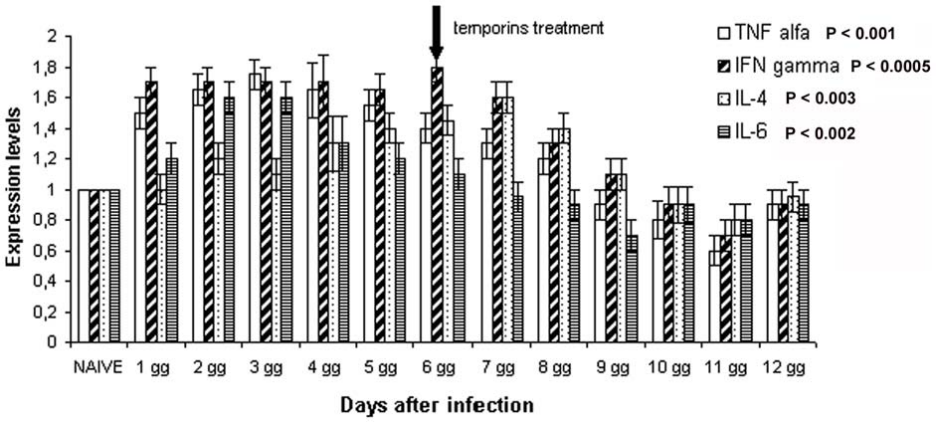
Expression levels of cytokine genes in mice infected with *S.enterica* serovar Paratyphi B (10^5^ CFU/mouse) and 6 days lose treated with temporins (35/75 pool). Each hystogram represents the mean value measured in 2 mice, each one tested in triplicate.

Treated mice, compared to the untreated ones, showed lower levels of the pro-inflammatory cytokines TNF-α and INF-γ and higher levels of the anti-inflammatory cytokines IL-4 and IL-6, indicative of a more balanced Th1/Th2 response (Figure 8). To study the anti-inflammatory activity of temporins under conditions that more closely reproduce bacterial infection in humans, the experiment was repeated using a small initial inoculum of bacteria. Mice were infected with *S. enterica* serovar Typhimurium (10^5^ CFU/mouse) and 6 days later treated with the 35/75 pool. The cytokine expression levels in the liver were monitored daily by RT-PCR. The experiment demonstrated that temporins bring the levels of the cytokines back to those of naïve mice within 5 days (Figure 9). The experiments on cytokines as described in this paragraph were repeated on mice infected with *S. aureus* and yielded very similar results.

**Figure 10:**
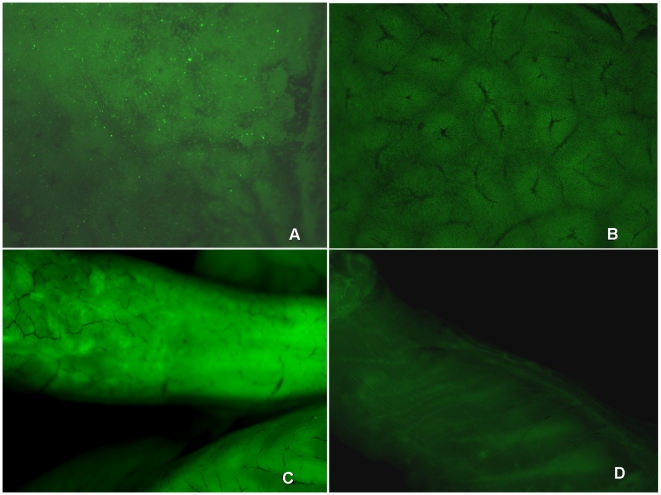
Mice infected with GFP-labelled bacteria were analysed using the Leica macrofluo instrument (Wetzlar, Germany) equipped with the Leica application suite 3.1.0 software. A: liver from mice infected with *S. enterica* serovar Paratyphi B; B: liver from mice infected with *S. enterica* serovar Paratyphi B and immediately treated with TA plus TB-YK; C: gastro instestinal tract from mice infected with *S. enterica* serovar Paratyphi B; D: gastro instestinal tract from mice infected with *S. enterica* serovar Paratyphi B and immediately treated with TA plus TB.

Mice were infected with a sub-lethal dose of *Salmonella GFP* 10^6^ CFU/mouse and immediately treated with the 35/75 pool. 24 hours later, the animals were sacrificed and intestine and liver were extracted. Observation of the organs at the Macrofluo microscope revealed a reduction in the fluorescence of the treated mice, as compared to untreated mice, which further confirms the efficacy of the pool 35/75. (Figure 10)

## Discussion

The antimicrobial activity of temporin A and a temporin B analogue has been investigated in vitro and in vivo. Results reported here highlight how small alterations of the native temporin B primary structure can influence dramatically its properties. Addition of two extra positive charged lysine residues at the N-terminus conferred to the analogue (TB-YK) a strong synergism with the native temporin A (Table 2).

In order to get details on the secondary structure of the peptides and to demonstrate that addition of the βAβAKK tail in the fluorescent derivatives does not influence the secondary structure of the peptides, CD analysis was carried out on TB and TB-YKβA in sodium phosphate buffer pH 7.4 and in sodium phosphate plus sodium dodecyl sulfate (SDS) buffer pH 7.4. The SDS is widely used to mimic the negatively charged bacterial membrane. The CD spectra obtained show that TB and TB-YKβA do not have a defined secondary structure in the absence of SDS, while they assume an α helical conformation in SDS. These results suggest that: a) addition of the sequence βAβA KK at the N-terminus of TB does not alter the secondary structure of the peptide and b) both TB and TB-YKβA assume a defined secondary structure when interact with membranes. Furthermore, a comparison of the CD spectra of temporin B described here and temporin A reported in the literature indicates that the peptides have both the same conformation [15].

Alterations of the bacterial membrane for Gram positive and Gram negative bacteria and the effects on vitality and morphology of cells were investigated by electron microscopy and fluorescence light microscopy. Treatment of both *S. aureus* A170 (Figure 2A–B) and *S. enterica* serovar Paratyphi B (Figure 2C–D) with combinations of TA and TB-YK results in severe perturbation of the membrane, as demonstrated by Figure 2 in which holes in the membranes are clearly visible. Analogous results are obtained visualizing the bacterial membrane by fluorescent light microscopy after treatment of the *S.aureus* A170 and *S. enterica* serovar Paratyphi B with combinations of TA-Rho and TBFluo. (Figure 3). When the same bacteria are treated with the fluorescent dyes (Rhodamine and fluoresceine), no damage is evident.

The inhibition zone assay confirms that the combination of TA and TB-YK is more potent than the single peptides: the inhibition diameter of the bacterial growth on the plate is wider when the TA and TB-YK are pooled than when peptide each is tested.

Studies of the haemolytic activity of various TA/TB-YK combinations carried out on mouse red blood cells revealed that the pool 35/75 had no haemolytic effect, unlike the wt TA and TB and other temporins [27], [28].

The 35/75 pool, given concurrently with a lethal dose of bacteria (gram positive or negative), rescued 100% of the animals (Figure 5). More importantly, the same pool, administered one week after experimental infection with a sublethal dose of bacteria, sterilized 100% of the animals within 3–6 days (Figure 6). Sterilization was attained in the blood and the target organs (liver and GI tract in the case of infection with *S. enterica* serovar Paratyphi; the kidneys in the case of infection with *S aureus*) without bacterial rebound. In none of the experiments did the 35/75 pool cause adverse effects due to rapid lysis of bacteria. These results were observed in the case of infection with gram positive (*S aureus*) or gram negative (*S enterica* serovar Paratyphi) pathogens. The general significance of these data invites an objective assessment of A and B temporins as antimicrobials.

Temporins displayed also a remarkable anti-inflammatory activity in vivo. Sepsis, an overzealous inflammatory response to infection, is increasingly common and frequently lethal. In the USA the incidence of sepsis is 240 per 100 000 population and the fatality rates range from 25% to 70%, depending on severity and comorbidity [29]. Also, sepsis from gram positive bacteria has matched that of sepsis from gram negative bacteria [30]. In this context, the anti-inflammatory activity displayed by the 35/75 pool represents a valuable result, especially since directed against gram positive and gram negative bacteria. As to the mechanism leading to the control of inflammation, it appears important the role of IL-6, a cytokine known to control the level of pro-inflammatory cytokines (TNF-α and INF-γ) without interfering with the expression of the anti-inflammatory cytokine IL-4 [31]. High doses of bacteria commonly administered to experimental animals cause rapid lysis by complement. This leads to a potential model of intoxication with endotoxins rather than a true model of sepsis [32]. The approach adopted in this study is exempt from this drawback since mice were infected with a small initial bacterial inoculum.

In the course of this study, a large number of mice were infected with different bacterial species. In no case bacterial colonies resistant to the 35/75 pool were recovered. In vitro experiments aimed at isolating mutants resistant to the 35/75 pool were also inconclusive. These results suggest that the bacterial membrane (the target of temporins) is a cellular component that bacteria cannot alter easily in order to gain resistance. This finding assumes great value in the light of the increasing incidence of antibiotic resistance.

In conclusion, the article provides evidence that temporin B, following limited alterations of its primary structure, acquires the capacity to act in synergism with temporin A and to exert antimicrobial and anti-inflammatory activity in vivo against gram positive and gram negative bacteria. The animal model described closely mimics bacterial infection in humans. This model offers the possibility to test in a preclinical setting the true potential of temporins as antimicrobial and anti-inflammatory agents.
